# Reformulating Pro-Oxidant Microglia in Neurodegeneration

**DOI:** 10.3390/jcm8101719

**Published:** 2019-10-17

**Authors:** Juan García-Revilla, Isabel M. Alonso-Bellido, Miguel A. Burguillos, Antonio J. Herrera, Ana M. Espinosa-Oliva, Rocío Ruiz, Luis Cruz-Hernández, Irene García-Domínguez, María A. Roca-Ceballos, Marti Santiago, José A. Rodríguez-Gómez, Manuel Sarmiento Soto, Rocío M. de Pablos, José L. Venero

**Affiliations:** 1 Department of Biochemistry and Molecular Biology, Faculty of Pharmacy, University of Seville, 41012 Seville, Spain; juangare79@gmail.com (J.G.-R.); isaalobel@gmail.com (I.M.A.-B.); maburguillos@us.es (M.A.B.); ajherrera@us.es (A.J.H.); anaespinosa@us.es (A.M.E.-O.); rruizlaza@us.es (R.R.); luiscruzhernandez1994@gmail.com (L.C.-H.); marian.roca.ceballos@gmail.com (M.A.R.-C.); msantiago@us.es (M.S.); msarmiento@us.es (M.S.S.); depablos@us.es (R.M.d.P.); 2 Institute of Biomedicine of Seville (IBIS)-Hospital Universitario Virgen del Rocío/CSIC/University of Seville, 41012 Seville, Spain; rodriguez@us.es; 3 Departament of Medical Physiology and Biophysics, Faculty of Medicine, University of Seville, 41009 Sevilla, Spain

**Keywords:** microglia, oxidative stress, neurodegeneration, inflammation, disease-associated microglia (DAM), ROS, RNS

## Abstract

In neurodegenerative diseases, microglia-mediated neuroinflammation and oxidative stress are central events. Recent genome-wide transcriptomic analyses of microglial cells under different disease conditions have uncovered a new subpopulation named disease-associated microglia (DAM). These studies have challenged the classical view of the microglia polarization state’s proinflammatory M1 (classical activation) and immunosuppressive M2 (alternative activation). Molecular signatures of DAM and proinflammatory microglia (highly pro-oxidant) have shown clear differences, yet a partial overlapping gene profile is evident between both phenotypes. The switch activation of homeostatic microglia into reactive microglia relies on the selective activation of key surface receptors involved in the maintenance of brain homeostasis (a.k.a. pattern recognition receptors, PRRs). Two relevant PRRs are toll-like receptors (TLRs) and triggering receptors expressed on myeloid cells-2 (TREM2), whose selective activation is believed to generate either a proinflammatory or a DAM phenotype, respectively. However, the recent identification of endogenous disease-related ligands, which bind to and activate both TLRs and TREM2, anticipates the existence of rather complex microglia responses. Examples of potential endogenous dual ligands include amyloid β, galectin-3, and apolipoprotein E. These pleiotropic ligands induce a microglia polarization that is more complicated than initially expected, suggesting the possibility that different microglia subtypes may coexist. This review highlights the main microglia polarization states under disease conditions and their leading role orchestrating oxidative stress.

## 1. Microglia: Origin and Roles in Health and Disease; Triggering Receptors Expressed on Myeloid Cells-2 (TREM2) Significance

A century ago, del Rio-Hortega described, for the first time, the existence of cells that he considered “the third element of nerve centers” [1] and later termed ‘microglia’ [2]. These are immune cells, macrophages residing in the central nervous system (CNS). Microglial cells derive from progenitors that originate in the yolk sac [3,4] and later proliferate and migrate to the CNS [5], continuously monitoring the brain parenchyma to maintain the correct homeostasis of the nervous tissue [6].

Microglial cells, reactive oxygen species (ROS), and pathological aggregated proteins have been long suspected to belong to a detrimental loop intrinsically involved in neurodegeneration. However, the strongest evidence about the role of microglia in neurodegeneration has been obtained by different genome-wide association studies (GWAS). These studies have identified different risk genes that are either enriched or uniquely expressed in immune cells, including microglia [7,8,9]. Representative examples are triggering receptor expressed on myeloid cells-2 (*Trem2*), *Cd33*, *Cr1*, *Mef2c*, members of the MS4A family, and the HLA locus, which are Alzheimer’s disease (AD) risk genes. These studies clearly point at microglia as leading actors in disease progression at very early stages of neurodegeneration [9]. From the broad array of immune-associated genes related to neurodegeneration, *Trem2* certainly emerges as a critical factor in governing microglia activation states. In an effort to characterize molecular systems associated to AD, Zhang et al. [10] performed a whole-genome gene expression, profiling and genotyping data in hundreds of samples from late-onset AD (LOAD) patients and aged-matched controls subjects [10]. This study identified a significant number of modules ascribed to different functional categories and cellular phenotypes. Rank ordering of the most significant molecular networks identified the immune/microglia module, including tyro protein kinase binding protein (TYROBP; also known as DAP12), as the highest ranking in terms of regulatory strength and differential expression [10]. TYROBP is the adaptor protein binding partner of TREM2, which also binds a large number of immune receptors including TREM1, CLEC7A, SIRPβ, PILRβ, and NKp44 [9,11]. The study by Zhang et al. anticipated the importance of TREM2 in triggering microglia activation associated to neurodegenerative diseases. Since the cloning of TREM2, the attention on this receptor in microglia functioning under homeostatic and neurodegenerative conditions has been increased exponentially (for reviews see [12,13]). Two independent studies identified a rare variant of TREM2 (R47H) as a strong risk gene of AD [14,15]. Following this, another variant (R62H) was further identified, thus, supporting the important role of TREM2 in neurodegeneration. Since TREM2 is uniquely expressed by microglia in the CNS [13], further elucidation of TREM2-dependent roles of microglia under disease conditions has become a priority in the field. In this review, we will focus not just on TREM2 but on other critical receptors involved in microglia polarization such as toll-like receptors (TLRs), historically linked only in the proinflammatory activation of microglia (highly pro-oxidant).

## 2. Sensing the Disease-Associated Environment: TLRs and TREM2, Main Drivers of Microglia Polarization

### 2.1. Toll-Like Receptors (TLRs)

The mammalian TLR family consists of 13 members that recognize specific patterns from different microbial components called pathogen-associated molecular patterns (PAMPs). Other endogenous molecules that are constitutively expressed and released upon injury, known as danger-associated molecular pattern molecules (DAMPs) [16], are also clearly involved. TLRs are constitutively expressed in different brain cell types, including microglia [17]. TLRs are either localized in the cell surface or in intracellular compartments such as the endoplasmic reticulum (ER) and endosome. Each member of the TLR family is constituted by three different structures: (i) an ectodomain with a horseshoe-like structure with leucine-rich repeats (LRRs) for PAMPs/DAMPs recognition; (ii) a transmembrane domain; and (iii) a cytoplasmic Toll/IL-1 receptor (TIR) domain that initiates downstream signaling. Upon recognition of their respective PAMPs/DAMPs, TLRs interact as a homo- or heterodimer, together with a co-receptor. After that, TLRs recruit TIR domain-containing adaptor proteins such as MyD88 and TRIF, which trigger different signal transduction pathways that end with the activation of NF-κB, IRFs, or MAP kinases [18]. Apart from the well-documented function for TLRs as the first barrier against deleterious stimuli of a different nature by triggering the innate immune response [19], several new roles have been assigned to TLRs [20]. In the context of neurodegenerative diseases, where a sterile inflammation occurs [16], different endogenous disease-related ligands may bind to and activate TLRs, such as amyloid β (Aβ) [21], α-synuclein (α-syn) [22], high-mobility group box protein 1 (HMGB1) [23], galectin-3 (GAL3; coded by *Lgals3*) [24,25], etc. After ligand recognition, TLRs assemble and activate different downstream signaling pathways, which activate several immune responses. For instance, TLR activation is necessary for priming inflammasome and to induce a second signal (e.g., Aβ aggregates in AD) that will lead to the generation of the inflammasome complex [26].

In the context of ROS production, TLR stimulation does also “prim” the activation of NADPH oxidases (NOX) [27]. The NOX complex consists of several proteins that are recruited and activated in the cell membrane in response to TLR engagement [28]. For instance, p47phox is translocated into the membrane and phosphorylated at Ser345 via ERK pathway in lipopolysaccharide (LPS)-treated microglia [29]. The role of activated NOX2 has been proven in several models of neurodegenerative diseases. Thus, microglia in AD subjects displayed activated NOX2, resulting in the formation of ROS that are toxic to neighboring neurons [30]. Gene deletion of gp91phox confers protection in animal models of Parkinson´s disease (PD, MPTP model) [31,32] Huntington’s disease (HD) [33], and LPS-induced neuronal damage [34]. Furthermore, TLR activation induces the expression of the GTP/GDP exchange factor Vav, which promotes nucleotide exchange over Rac2, a pivotal protein during the oxidative burst in neutrophils and macrophages [35].

The high immune-related oxidative burst initiated by TLR is normally used as an “arsenal” to fight infection [36]; however, it may also play a signaling role in immune cells. For instance, the small acute oxidant burst induced by TLR activation mediates the rearrangements of TLRs into the lipid rafts in the membrane of macrophages [37,38]. Additionally, their role has been described during the formation of TRAF6 and ASK1 complex [39], both necessary for TLR-mediated inflammatory responses [40].

### 2.2. TREM2

TREM2 is a fundamental receptor in sensing the environment under disease conditions. It is uniquely expressed in cells of the myeloid lineage. The extracellular domain of TREM2 belongs to the immunoglobulin superfamily and recognizes an extensive range of polyanionic molecules including LPS, glycosaminoglycans, phospholipid, and DNA [41,42,43]. Upon ligand binding, TREM2 is known to interact with two different adaptor proteins: DAP12 and DAP10. The intracellular domain of DAP12 activates spleen tyrosine kinase (SYK), activating downstream pathways that regulate survival, migration, proliferation, activation, and phagocytosis [13]. On the other hand, interaction of TREM2 with DAP10 leads to phatidylinositol 3-kinase (PI3K) recruitment, which triggers a phosphorylation cascade that promotes Ca^2+^ mobilization, integrin activation, cytoskeleton rearrangement, mechanistic target of rapamycin (mTOR) and mitogen-activated protein kinase (MAPK) signaling, and activation of energetic metabolism [13]. How exactly each ligand activates different and specific signaling pathways of the TREM2 response remains unclear. Mutations and single nucleotide polymorphisms (SNPs) in the TREM2–DAP12 axis have been related to a variety of neurodegenerative disorders such as Nasu–Hakola disease, frontotemporal dementia, AD, PD, amyotrophic lateral sclerosis (ALS), and essential tremor (for reviews see [13,44,45]). Interestingly, the majority of the TREM2 disease-linked mutations occur in their extracellular Ig-like V-type domain [46]. These findings, together with the role of TREM2 in driving the disease-associated microglia (DAM) phenotype, strongly link the TREM2 signaling to the pathology of neurodegenerative diseases. Yet, the mechanism(s) where TREM2 is implicated in pathology progression has not been totally clarified. Indeed, it is unclear whether TREM2-related microglial signaling pathways are either protective or deleterious in neurodegeneration. This controversy may be related to the ambiguous role of TREM2 in modulating oxidative stress. Uncovering the molecular pathways activated by TREM2 during microglia activation under disease conditions and how this microglia polarization may lead to harmful oxidative stress will help to develop novel therapeutic approaches to treat neurodegenerative diseases.

## 3. Microglia Subtypes Under Conditions of Neurodegeneration

Historically, microglia polarization states were simplified in two opposite phenotypes termed proinflammatory M1 (classical activation) and immunosuppressive M2 (alternative activation; also known as tumor-supportive phenotype). However, recent massive transcriptomic studies have clearly demonstrated the oversimplification of the M1/M2 microglia polarization states and the existence of different microglia subtypes under disease conditions is now evident.

### 3.1. Classical M1-Like and M2-Like Phenotypes

The classical microglia M1 or proinflammatory phenotype is induced after LPS/(IFN)-γ treatments and upregulates inducible nitric oxide synthase (iNOS) and NF-κB nuclear translocation [47]. This activation leads to strong activation of different proinflammatory cytokines, including tumor necrosis factor (TNF)-α, interleukin (IL)-1β, NLRP3 (the best known inflammasome), FLIP (survival protein), etc. The M1 phenotype has been classically linked to oxidative and nitrosative stress generation [47]. It is noteworthy to mention that sustained proinflammatory activation of microglia has long been considered neurotoxic [48].

On the other side, alternative activation (M2-like) of microglia is achieved upon treatment with IL-4 or IL-13 and triggers upregulation of anti-inflammatory genes including arginase-1 (source of proline and polyamines), mannose receptor (CD206), YM1, and FIZZ. This activation pathway is considered to contribute to tissue repair and extracellular matrix reconstitution [49].

**Figure 1:**
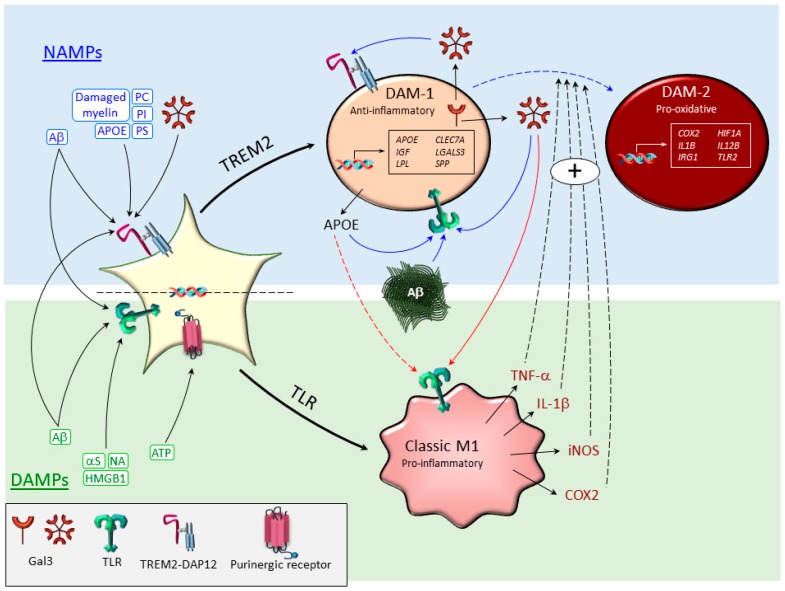
Potential cross-interactions between different disease-associated microglia polarization subtypes. It is well established that microglia sense the disease environment through different pattern recognition receptors (PRRs). Two illustrative examples are toll-like receptors (TLRs) and triggering receptors expressed on myeloid cells-2 (TREM2). Specific ligands of PRRs are different danger-associated molecular patterns (DAMPs), including aggregated proteins (amyloid β, Aβ, and α-synuclein, αS); high-mobility group box protein 1 (HMGB1); nucleic acids (NA); and ATP. From these, Aβ and HMGB1 are believed to activate TLRs, thus driving microglia to a M1-proinflammatory phenotype, which is highly pro-oxidant. More recently, the term neurodegeneration-associated molecular patterns (NAMPs) has been introduced to highlight endogenous disease-associated ligands of TREM2. Examples of NAMPS include phosphatdyil serine (PS), present in apoptotic cells and glycolipids sphingomyelin and sulfatide derived from damaged myelin; Aβ; several lipoproteins like apolipoprotein E (APOE); and negatively charged phospholipids like phosphatidylinositol (PI) and phosphatidylcholine (PS). TREM2 signaling is suggested to drive the disease-associated microglia (DAM) phenotype, leading to downregulation of microglia homeostatic genes (not shown) and strong upregulation of DAM genes, including *Apoe*, *Lgals3* (galectin-3; GAL3), *Clec7a*, etc., and thus, driving microglia to an anti-inflammatory (DAM-1) phenotype. From these, APOE and GAL3 can be released by reactive microglia and govern microglia immune responses. The possibility exists that GAL3 and APOE, along with other endogenous ligands like Aβ, drive TLR-associated signaling (solid blue arrows) further in either pro-oxidative DAM phenotypes (DAM-2; dashed blue arrow) or classic M1 proinflammatory microglias (red arrows). In addition, different classical microglia proinflammatory mediators like tumor necrosis factor (TNF)-α, interleukin (IL)-1β, inducible nitric oxide synthase (iNOS), and cyclooxygenase-2 (COX2) may affect the DAM phenotype, which may thus evolve into a pro-oxidative DAM phenotype.

**Figure 2:**
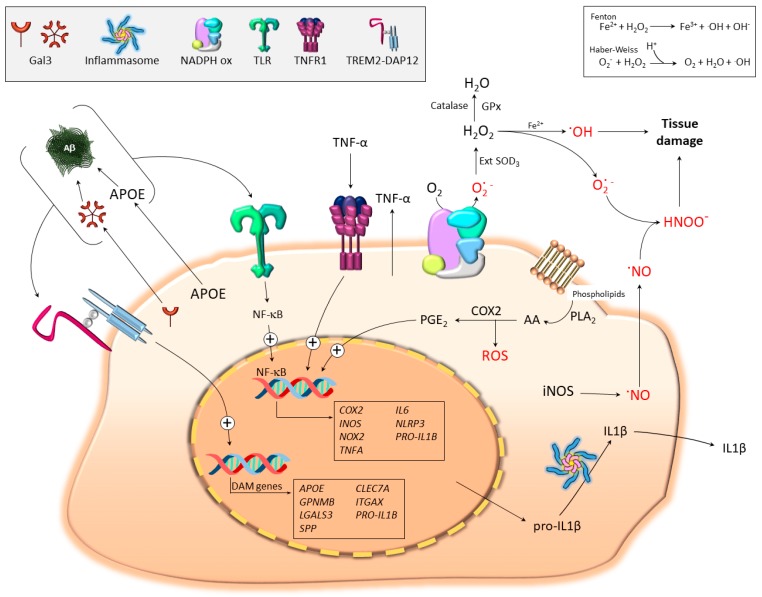
Pro-oxidant microglia under disease conditions. TLR signaling drives NF-κB activation and transcription of proinflammatory and pro-oxidant molecules including iNOS, COX2, NADPH oxidase (NOX2), TNF-α, and pro-IL-1β. Assembly of NOX2 and ulterior activation constitutes an important source of superoxide anion and subsequent formation of radical oxygen (ROS) and nitrogen species. The figure illustrates how extracellular superoxide dismutates to H_2_O_2_ through the extracellular activity of superoxide dismutase (SOD) 3 and formation of hydroxyl radicals through the Fenton and Haber–Weiss reactions. Alternatively, superoxide anion may react with nitric oxide (NO) to form the highly toxic reactive peroxynitrites. The figure also illustrates the important role of COX2 in generating ROS. Thus, phospholipase A2 (PLA2) supplies arachidonic acid (AA) to COX2 for prostanoid biosynthesis (PGE2) along with ROS. NF-κB activation also leads to NLRP3 upregulation (the main inflammasome component). Upon appropriate stimulation (not shown; examples include K^+^ efflux or cathepsin release from damaged lysosomes), NLRP3 assembles a multiprotein platform resulting in caspase-1/caspase-8 activation and subsequent cleavage of pro-IL-1β into an active mature form (IL-1β). The figure also illustrates how different multivalent ligands, including Aβ, galectin-3 (GAL3), and APOE, may drive both TLR- and TREM2-signaling pathways. The switch from homeostatic to disease-associated microglia (DAM) is believed to be TREM2-dependent and it is accompanied by strong upregulation of different genes including GAL3 and APOE. These proteins can be released to the extracellular space, which together with Aβ and other DAMPS, may bind to and activate TLRs and trigger the microglia pro-oxidant response.

Since arginase-1 and iNOS compete for the same substrate (arginine; Arg), their equilibrium during disease will dictate nitric oxide (NO) production and subsequent oxidative stress. However, owing to the bigger complexity of the coexistence of a broad panel of microglial phenotypes during the progress of disease, the real existence of M1-like and M2-like phenotypes in microglial cells has already been a source of needed debate [50]. Since this review is devoted to discussing important aspects of microglia-associated oxidative stress and neurodegeneration, it is important to define particular microglial states that may be involved. Without any doubt, the microglia proinflammatory phenotype is pro-oxidant, and a strong induction of iNOS is a hallmark of this important phenotype [47] (see Section 4.2 of this review) (Figure 1 and Figure 2). Transcriptional profiles of isolated microglia in response to systemic LPS have been recently generated in an effort to understand the proinflammatory phenotype in vivo [51,52]. These studies highlight key differences between proinflammatory and DAM phenotypes. Findings showed that the LPS-induced microglia phenotype was not associated to DAM-like features such as TREM2/DAP12 or apolipoprotein E (APOE) upregulation, [51,52], although different DAM genes were upregulated (e.g., *Itgax* (CD11c), *Axl*, *Clec7a,* and *Ccl2)* (see Section 3.2). However, highly upregulated genes in response to LPS were associated with an oxidative stress response, especially *Ptgs2* (COX2) and *Cybb* (NOX2) (major source of free radicals, see Section 4.2 and Figure 2) and classical proinflammatory genes like *Il1β*, *Tnfα,* and *Ccl2* [51,52].

### 3.2. Disease-Associated Microglia (DAM)

The advent of massive transcriptomic analysis at the single cell level has helped to uncover different microglia states associated with controling homeostasis [51,53,54,55], aging [51], and disease-associated conditions [56,57,58,59]. Holtman et al. [56] analyzed transcriptional profiles of isolated microglia from different mouse models of aging and different diseases using weighted correlation network analysis (WGCNA)-based meta-analysis to determine the transcriptional signature and hub genes of different primed microglia phenotypes. In this study, after analyzing different disease-associated conditions like AD, ALS, and physiological and accelerated aging, they found a similar transcriptional network. Subsequent studies by Amit and colleagues using single-cell RNA analysis of brain immune cells identified a microglia subpopulation showing a unique molecular signature in 5xFAD mice (a transgenic mouse model of AD), which they named “disease-associated microglia” (DAM) [57]. In this study, two microglia clusters (groups II and III) were observed in 5xFAD mice but not in wild-type mice. They were characterized by (i) downregulation of key homeostatic genes including *P2ry12, Cx3cr1,* and *Tmem119* and (ii) upregulation of selective genes (e.g., *Apo3, Ctsd, Lpl, Itgax*, *Clec7a, Tyrobp* and *Trem2)*. Based on the degree of upregulation of DAM genes, the authors hypothesized that group II is an intermediate state between homeostatic microglia and group III (DAM), which was associated with microglia clustering Aβ plaques [57]. Mechanistically, the authors concluded that transition from group II microglia to DAM was TREM2-dependent, whilst transition from homeostatic microglia to group II was TREM2-independent. The relevance of this finding is the fact that many DAM genes, especially *TREM2*, have been found as risk genes of AD and other neurodegenerative conditions.

In an independent study, Butovsky and colleagues also performed transcriptomes of isolated microglia during different conditions including aging, mouse models of AD (APP/PS1), ALS (SOD1^G93A^), and multiple sclerosis (MS) (experimental autoimmune encepahalomyelitis; EAE) [51]. Again, a similar microglia molecular response was found in the different models, exhibiting loss of homeostatic genes (e.g., *P2ry12*, *Tmem119*, *Gpr34*, *Csf1r*, *Hexb,* and *Mertk*), together with upregulation of inflammatory genes including *Spp1*, *Itgax*, *Lgals3*, *Axl*, *Clec7a*, *Ccl2,* and *Apoe* [51]. Although this microglia phenotype was named microglia neurodegenerative phenotype (MGnD), it shares many features with DAM [57], and hence, we will follow this nomenclature to avoid unnecessary confusion. Interestingly, microglia APOE upregulation correlated with disease progression in EAE, SOD1, and APP/PS1 models, thus raising the possibility that DAM could be neurotoxic [51]. Interestingly, APOE is believed to play a major role in the generation of the DAM phenotype, as *Apoe* deletion largely prevented upregulation of different DAM genes including *Clec7a*, *Gpnmb,* and *Lgals3* [51]. Gene targeting of *Trem2* overall prevented the switch from homeostatic to DAM microglia, and ameliorated the neuronal loss following facial nerve axotomy in SOD1 and APP/PS1 models [51].

In tau models, TREM2 is increased at late stages [60,61] and its inhibition protects against tau-induced brain neurodegeneration [61]. Since chronic neurodegenerative diseases are persistent in terms of neuronal cell death, the concept that microglia act as a double-edged sword in neurodegenerative disease has been lately a constant debate in the field [62]. Among the different DAM genes, glycoprotein nonmetastatic melanoma protein B (Gpnmb; also known as osteactivin) is a very interesting candidate in driving TREM2-dependent anti-inflammatory effects. Its importance is supported by the existence of Gpnmb gene mutations associated with increased risk of developing PD [63]. Regarding the inflammatory-related function of GPNMB, there is deep evidence about its role as a negative regulator of inflammatory process [64,65,66,67]. In addition to its potential anti-inflammatory role, it has been described that GPNMB is related to the autophagic-lysosomal pathway, involved in lysosomal stress and in the degradation of cellular debris [68,69].The fact that GPNMB is overexpressed in a variety of neurodegenerative diseases, its key role as anti-inflammatory, and its relation with the autophagic-lysosomal pathway suggest that the normal function of this protein is essential for keeping homeostatic brain status. Therapeutic approaches modulating GPNMB function might raise potential treatments for neurodegenerative diseases.

A key question is to understand the pro-oxidant role of DAM. It is paradoxical that absence of TREM2 may be protective in some paradigms [51,61] and exacerbate neuronal damage in others. Thus, absence of TREM2 in AD mice, which halts the switch from homeostatic microglia to DAM, leads to increased neuritic dystrophies associated with Aβ plaques [70,71]. The same is true in human carriers of the R47H TREM2 mutant form [12]. Similarly, the impairment of native TREM2 activity associated with its R47H variant seems to facilitate the formation of tau aggregates in the vicinity of neuritic plaques, owing to the reduction of microglia activation [72].

The microglia polarization state relies on selective activation of microglia surface receptors by a broad nature of ligands. Historically, the microglia field has consistently used the terms PAMPs and DAMPs, which are recognized by pattern recognition receptors (PRRs) (Figure 1) [7]. Prototypical PRRs include the TLRs (see 2.1). Other examples of PRRs are the NOD-like receptors, C-type lectin receptors, and the receptor for advanced glycation end-products (RAGE) [73]. Recently, the term neurodegeneration-associated molecular pattern (NAMPs) has been suggested, which includes an array of danger signals commonly present in different neurodegenerative diseases and recognized by different receptors (including TREM2) to switch from homeostatic microglia to DAM (Figure 1) [74]. The family of NAMPS includes recently identified TREM2 ligands. The typical examples include phosphatidyl serine present in apoptotic cells and glycolipids sphingomyelin and sulfatide derived from damaged myelin (Figure 1). However, new interesting candidates could be products associated with Aβ plaques, including Aβ itself [75,76], several lipoproteins like APOE and CLU/APOJ [77], phosphatidylinositol, and phosphatidylcholine [43,78]. Besides, TREM2 has been found to bind different bacterial products, including those from Gram-negative, whose membrane contains LPS [45]. More recently, we have demonstrated an important role of GAL3, a carbohydrate-binding protein in AD pathology, and more important, its ability to bind to and stimulate TREM2 (Figure 1) [79]. However, GAL3 stimulates not just TREM2 but also other fundamental microglial receptors, including TLR4 [24] and insulin-like growth factor 1 receptor (IGFR1) [80]. GAL3 has been shown to be important in driving the proinflammatory activation of microglia in response to fibrillar Aβ [79]. Since GAL3 can be released by reactive microglia [24,79], there is a strong possibility that GAL3 may act as an endogenous candidate to drive both TLR- and TREM2-dependent signaling pathways (Figure 1 and Figure 2). Indeed, this feature is not restricted only to GAL3 since there are other exciting examples, including APOE and Aβ (Figure 2). Thus, *Apoeε4* allele, one of the strongest gene risk factors in AD, has been associated with strong proinflammatory stimulation of the human innate immune response through TLR activation [81].

In addition, microglia activation in response to Aβ has been previously shown to involve both TLR signaling and NOD-like receptors [26,82,83]. In fact, amyloid plaque compaction involves a chronic exposure of microglia to Aβ [83,84], which has been shown to induce the release of microglial proinflammatory and pro-oxidant factors that mediate neuroinflammation, a process that can be detrimental to the nervous tissue. Altogether, we could certainly expect that different microglia receptors play significant roles in driving microglia heterogeneous responses under disease conditions, including proinflammatory/pro-oxidant components (Figure 2). In the same vein, a recent study has applied WGCNA to existing transcriptomic datasets from CNS immune cells and analyzed three AD modules, including 5xFAD, 5xFAD/TREM2 knock-out (KO), and APP/PS1 [85]. This study identified two well-defined modules; a proinflammatory pro-oxidant module, including *Ptgs2*, *Il12b*, *Tlr2*, *Hif1α*, *Il1β*, and *Irg1,* and an anti-inflammatory module, including anti-inflammatory and phagocytic genes like *Igf1*, *Dpp7*, *Apo3*, *Spp1,* and *Lpl* [85,86]. Importantly, these two modules are closely related and are very different from the homeostatic microglia [85]. Both modules exhibited DAM genes and they were partly TREM2-dependent, thus, supporting the existence of at least two well-defined DAM subtypes with a common origin [43]. In addition, transcriptomic analysis in brains from 24-month-old aged wild-type and TREM2 KO mice revealed that most significant genes in response to TREM2 deficiency were associated to the complement system, microglia, and the oxidative stress response *Cybb* (NOX2) and *Nos2* (iNOS) genes [87].

Lastly, different studies analyzing the presence of lipid peroxidation biomarkers, such as 4-hydroxynonenal (HNE), malondialdehyde, and intracellular ROS (see Box A1), revealed high levels in TREM2 KO mice, thus, supporting the view that TREM2 may also repress an oxidative stress response [87,88]. These studies are in line with those showing that activation of TREM2 modulates the TLR-induced inflammation, following a reduction in proinflammatory cytokine release and a decrease in ROS production (for review see [89,90]). However, the last referred studies were performed in vitro, which have been shown not to mimic the prototypical DAM phenotype demonstrated under disease conditions in vivo.

### 3.3. Necroptotic Microglia

Within the different potential pro-oxidant states of microglia, necroptotic microglia deserves special attention since they have been described as highly proinflammatory and immunogenic. Necroptosis, a particular form of cell death, can be induced in response to different cellular stimuli, including TNF-α, FAS ligand, TRAIL, IFNγ, ischemia-reperfusion injury, and double-stranded RNA (dsRNA). Caspase-8 and receptor interacting protein kinases (RIPK1 and RIPK3) are essential to engage the necrosome assembly in order to activate necroptosis. Depending on the experimental settings, RIPK1 may repress or induce necroptosis [91]. At the same time, necroptosis can follow a dependent or independent RIPK1 pathway. In either case, activation of RIPK3 is always involved. RIPK3 activation phosphorylates and activates mixed lineage kinase domain-like pseudokinase (MLKL), a typical feature of necroptosis. Activated MLKL causes destabilization of plasma membrane and cell death [91,92]. It has long been assumed that necroptosis is highly proinflammatory because of the release of DAMPs. However, recent data support the view that necrosome formation may trigger a robust, cell death-independent, proinflammatory response [93,94]. Indeed, necroptosis has also been observed in primary microglia linked to TNF-α production [95]. The occurrence of physiological necroptopsis of microglia has been recently demonstrated [96]. Interestingly, the levels of RIPK1 are increased in brain samples from individuals with LOAD and positively correlate with Braak stages [97,98], thus, supporting a major role of necroptosis in neurodegenerative diseases. Evidence from these studies and those provided by AD mice models suggest necroptotic microglia as one of the key players leading to neuroinflammation and neurodegeneration [99].

### 3.4. Dark Microglia

Tremblay and colleagues identified a distinctive microglia subtype associated with the aging process, chronic stress, and AD and referred it as dark microglia (DM). This specific microglia subtype exhibits typical features of oxidative stress processes like condensed, electron-dense cytoplasm and nucleoplasm and endoplasmic reticulum dilation, giving them a “dark” appearance at the electron microscopy [100]. Although the physiological significance of DM has yet to be determined, they are thought to be extremely active, exhibiting highly ramified and extremely thin processes [100]. These processes are often associated with synaptic clefts, encircling axon terminals, dendritic spines, and entire excitatory synapses processes [100]. DM are also abundant during postnatal development, when microglia diversity is especially evident [101], and highly express Cd11b, which together with CD68, form the complement receptor 3 involved in synaptic pruning [101]. In the APP/PS1 mice, DM are associated with Aβ plaques and express TREM2, thus, raising the possibility that DAM and DM might be somehow related. Indeed, DM are associated to neuronal dystrophies [100]. Both DAM and DM downregulate homeostatic genes like *Cx3cr1* and *P2ry12*. However, DM fail to express *Cd11c,* while DAM highly upregulate this marker [57]. Whether DM derive DM in APP/PS1 mice (about two-thirds of total microglia) [100], and the extremely high oxidative stress status of this subtype, highlights the potential contribution of microglia-derived free radicals in AD pathology.

## 4. Microglial Contribution to Oxidative Stress

### 4.1. The Basis: Free Radical Generation in Biological Systems

Oxygen can be viewed as a double-edged sword as we need it for energy generation but oxygen-derived free radicals may harm cells too. The superoxide anion radical (O_2_^•−^), hydrogen peroxide (H_2_O_2_), and the hydroxyl radical (^•^OH) are regular products of respiration and oxidation processes in an aerobic environment [102]. These reactive molecules and free radicals are named as ROS. At the same time, reactive nitrogen species (RNS) are a similar collective term that includes radicals like ^•^NO and nitric dioxide (^•^NO_2_), as well as nonradicals such as nitrous acid (HNO_2_) and dinitrogen tetroxide (N_2_O_4_), among others [103].

Free circulating transition metals and enzymes that catalyze ROS-generating chemical reactions, such as peroxidases, NOX, xanthine oxidase, lipoxygenases, myeloperoxidase, NOS, and COX, are relevant sources of ROS [104]. Indeed, the different microglia polarization states are inherently associated with upregulation, among others, of iNOS, COX2, and NOX2, whose persistent activation leads to aberrant free radical production and tissue damage (Figure 2).

Detoxification of ROS is fundamental for all cells to survive. Living organisms have developed a variety of defense mechanisms to provide a balance between generation and elimination of ROS. The antioxidant protection system of the cell can be divided into antioxidant molecules and antioxidant enzymes. The antioxidant molecules of the cell include endogenous substances such as glutathione (GSH), uric acid, ubiquinone (coenzyme Q), lipoic acid, and bilirubin [105]; food antioxidant molecules such as ascorbic acid (vitamin C); and tocopherols and tocotrienols such as α-tocopherol (vitamin E), carotenoids, and flavonoids [106]. The human organism is equipped with very efficient antioxidative enzymes, such as superoxide dismutase (SOD), catalase, glutathione peroxidase, and glutathione reductase [107]. The enzymes catalase and SOD are the major defenses against ROS (Figure 2).

Any imbalance between ROS production and antioxidant defense creates oxidative stress that leads to irreversible damage to macromolecules such as proteins, DNA, and lipids. In particular, aberrant production of ROS and RNS in the CNS has been described as the source of neuronal loss in AD [108], PD [109], and ALS progression [110]. Further understanding of how aberrant oxidative stress contributes to neuronal cell death is a determinant in developing novel strategies to treat CNS disorders.

### 4.2. Pro-Oxidant Microglia Profiling: iNOS, COX2, TNF-α, and IL-1β

As illustrated in Figure 2, microglia activation triggers multiple signaling pathways leading to significant upregulation of multiple genes, from which several of them are inherently associated woth oxidative stress. In this section, we will highlight most representative ones, including iNOS, COX2, TNF-α, and IL-1β.

#### 4.2.1. iNOS and Nitric Oxide (NO)

NO has long been considered one of the most relevant molecules produced in the body. This promiscuous and polyvalent molecule is a master regulator of several physiological functions (e.g., blood pressure, immune response, cell metabolism, and neuron communication). To date, soluble guanylyl cyclase (sGC) is the most accepted physiological receptor described for NO, involving important regulatory actions in cell morphology and motility [111], including microglial activation and migration [112].

NO possesses an extra electron that makes it highly chemically reactive [113]. It is clear that activation of microglia encompassing the different microglia polarization states may cause sustained RNS production and subsequent damage to healthy host cells [113] (Figure 2). NO is synthesized by three isoforms of NOS, and all of them synthesize NO by hydroxylating L-Arg to N-hydroxy-L-Arg (NOHA), which is then oxidized to L-citruline and NO. The three isoforms are: (i) NOS type I (NOS1, nNOS, or neuronal), located in specific neurons of the central and peripheral nervous system, [114]; (ii) NOS type II (NOS2, iNOS, or inducible), induced by immunological and inflammatory stimuli and also during brain aging and brain diseases; and (iii) NOS type III (eNOS, NOS3, or endothelial), localized in the vascular endothelium as well as in some central neurons, where it plays a key role in synaptic plasticity and blood flow [115].

As previously stated, peroxynitrite is part of the RNS family, originated principally by the enzyme NOX after the reaction of NO with superoxide radical (Figure 2). In addition, cross-talk between NOS and NOX pathways has been demonstrated in different experimental settings. For example, gangliosides induce the activation of microglia, the production of proinflammatory cytokines (TNF-α, IL-1β), and the upregulation of iNOS that were attenuated by the inhibition of the NOX system with diphenylene iodinium [116]. Indeed, it has been demonstrated that stimulation of microglia by different stimuli including LPS, IFNγ, TNFα, IL-1β, arachidonate, and ATP generate extracellular NO. This leads to subsequent acute stimulation of NOX, inducing rapid breakdown of all extracellular NO, which is concomitant to peroxynitrite formation [117,118]. This mechanism has been shown to be toxic for neurons [118] (Figure 2). Therefore, the interplay between iNOS and NOX seems to be essential for microglia-mediated neurodegeneration.

In order to counterbalance the presence of peroxynitrites within the cells, there are other molecules that are involved in the reduction of peroxynitrite levels, such as glutathione (GSH), vitamin C, and vitamin E [119]. In general, there are several ways peroxynitrites induce cell death, owing to their potent oxidizing properties: apoptosis, autophagy, parthanatos [120], and necroptosis (already discussed in Section 3.3). Despite evidence that links apoptosis and peroxynitrites, the mechanisms are still unclear. The N-methyl-D-aspastate receptor (NMDAR) is a glutamate receptor in charge of activating peroxynitrite-dependent apoptosis. Glutamate activates NMDAR, increasing the intracellular levels of calcium. This rise boosts the production of NO by nNOS and superoxide in the mitochondria, leading to superoxide and peroxynitrite production. Exogenous peroxynitrites trigger the c-Jun N-terminal kinase (JNK) and p38 MAP kinases, following mixed lineage kinase (MLK) [121].

On the other hand, peroxynitrite inhibits the PI3 kinase/Akt pathway. Peptides including tyrosine are the main targets for the nitration caused by peroxynitrite. One of them is the molecular chaperon heat shock protein 90 (HSP90). Nitration in residue 56 leads to activation of the purine receptor P2X7, with subsequent increases in intracellular calcium and caspase activation to ultimately trigger motor neuron apoptosis [122]. Another source of peroxynitrites occurs when the endoplasmic reticulum removes misfolded and aggregated proteins through autophagy by the unfolded protein response (UPR). When faulty proteins begin to accumulate, there is a calcium influx into the cytosol. This activates the synthesis of ROS and NOS (e.g., hydrogen peroxide, superoxide, NO, and peroxynitrite), generating more misfolded proteins. The presence of such oxidative stress provokes the binding of misfolded proteins to the chaperone glucose-regulated protein 78 (GRP78), triggering the UPR, and subsequently, ROS and NOS [123].

#### 4.2.2. Cyclooxygenase-2 (COX2)

Recent massive single cell transcriptomic studies of microglia have certainly distinguished *PTGS2* as one of the most upregulated genes in proinflammatory conditions [51]. *PTGS2* codify the COX2 enzyme, a very important player in the metabolism of arachidonic acid, which leads to the production of prostaglandins (PGs) (Figure 2).

Among the different isoforms of COX, COX2 is the inducible form rapidly expressed in several cell types in response to growth factors and different cytokines, such as IL-1β, IL-6, or TNF-α [124]. This isoform has emerged as the main source of prostanoid (PG) production in acute and chronic inflammatory conditions [125] and its implication in neurodegenerative diseases has been extensively studied. Potential harmful downstream effectors of COX2 are PGE_2_ and free radicals (see Figure 2). PGE_2_ could have deleterious effects in the brain through several mechanisms that include inflammation, oxidative stress, and excitotoxicity. However, PGs also show neuroprotective effects, acting then with a dual role in brain diseases. Therefore, the beneficial or detrimental role played by COX2 in inflammation and neurodegenerative brain pathologies is still controversial [125]. Taking into account the important role of COX2 in inflammation, numerous studies have evaluated its role in the development of neurodegenerative diseases, including PD, AD, ALS, and MS [125], as well as the potential neuroprotective effects of COX2 inhibitors. Although it is unlikely that upregulation of COX2 activity alone produces enough free radicals to account for the degree of oxidative damage associated with neurodegenerative diseases, it may be one of the main players for the synergistic effect that causes significant accumulative damage (Figure 2).

Importantly, microglial COX2 expression was reported in post-mortem SN from PD patients and animal models of PD [126]. Moreover, increased susceptibility to excitotoxicity in COX2-overexpressing neurons has been shown in several experimental models of PD [127]. Regarding AD, over the last 20 years several analyses of COX1 and COX2 expression in animal models and post-mortem brain tissues have provided a substantial but still controversial body of evidence pointing at the involvement of COX2 in AD [125].

#### 4.2.3. Tumor Necrosis Factor α (TNF-α)

TNF-α is a pleiotropic inflammatory cytokine that plays a critical role in diverse cellular events, including cell proliferation, differentiation, apoptosis, and necrosis [128]. Through binding to its two receptors, TNFα-R1 (p55) and TNFα-R2 (p75), and causing NF-κB nuclear translocation, TNF-α is a major mediator of both inflammation and immune response and has been described to be implicated in a variety of pathological inflammatory conditions and autoimmune diseases. The neurotoxic role of TNF-α both in vivo and in vitro has been clearly established [129,130]. Increased levels of soluble TNF-α have been found in neurodegenerative diseases such as ischemic stroke, AD, PD, ALS, and MS. In PD and AD, this was supported by polymorphisms associated with increased TNF-α production in post-mortem brain patients [131,132]. Supporting the role of TNF-α in neurodegeneration, an increase of NF-κB levels in PD patients´ samples and numerous experimental models have been described [133].

Many pieces of evidence point out that at least part of TNF-α neurotoxicity is mediated indirectly through microglial cells and that ROS and RNS released by microglia are central elements of that process [134]. 

In response to TNF-α, among other cytokines, the multi-subunit enzyme complex is assembled at the plasma membrane, producing high levels of superoxide extracellularly, which may either generate hydrogen peroxide (catalyzed by extracellular SOD) or react with NO to produce peroxynitrites (Figure 2) [117]. Activation of microglial NOX alone causes no acute neurotoxicity, [118] but plays an important role in regulation of microglial activity itself in an autocrine manner. These effects include stimulation of microglial proliferation [135], production of TNF-α and IL-1β [136,137], and expression of iNOS [137].

#### 4.2.4. Interleukin 1β (IL-1β)

IL-1β belongs to the interleukin family with paracrine roles, leading to local inflammation and infiltration of other immune cells [138]. IL-1β is synthetized as zymogen in response to several PAMPs that are recognized by PRRs [139]. The synthesis of IL-1β can be divided into two steps. Firstly, a ′priming stag′ triggers the transcriptional machinery including pro-IL-1β. Later, a second stimulus is needed for the formation of the macromolecular platform called inflammasome (Figure 2). Inflammasome assembling includes the recruitment and activation of caspase-1 leading to pro-IL-1β cleavage into its active form (canonical inflammasomes) [140]. Examples of canonical inflammasomes include nucleotide-binding oligomerization domain-like receptors (NLR)P1, NLRP3, NLRC4, AIM2, and pyrin [141]. Among these five, NLRP3 is the main and most studied inflammasome, but other noncanonical inflammasomes and different effector caspases have emerged in recent years [142].

IL-1β ranks among the 20 most overexpressed genes in DAM microglia [51,57]. Implication of IL-1β in neurodegenerative diseases is well known because of the important role of neuroinflammation in these pathologies as well as well as the importance of oxidative stress. However, implication of oxidative stress in IL-1β production is still not fully understood. So far, convincing evidence for this interaction has been determined in NLRP3 inflammasome, suggesting mitochondria as the source of ROS that trigger inflammasome assembly [143]. Indeed, different studies support an important role of ROS in inflammasome activation during neurodegenerative diseases, which may act in conjunction with other signals like pathological protein aggregates. Heneka and colleagues studied the key role of NLRP3 inflammasome in AD [26] and the potential mediation of Aβ-induced ROS production [144]. In fact, inflammasome formation may exacerbate AD pathology through microglia release of ASC specks that promote Aβ cross-seeding [145]. In PD, mitochondrial dysfunction and oxidative stress have traditionally been associated with the pathogenesis of the disease. Recently, mitochondrial dysfunction along with ROS production has been proposed as a mild priming signal but a strong second signal for inflammasome formation in microglia [146]. This role of ROS supports the idea of α-syn as an inflammasome activator since PD patients showed unequivocal signals of inflammasome assembly [147]. The importance of pathological α-syn in inflammasome activation has already been thoroughly described [7].

IL-1β and inflammasome have been implied in other neurodegenerative diseases like ALS or virus infection. Poor implication of oxidative stress has been described in these pathologies, but recently, importance of peroxynitrites has emerged as the trigger of inflammasome in ALS disease [148]. Altogether, IL-1β seems to play an important role as a final effector of oxidative stress during neurodegeneration. Understanding the mechanisms linking oxidative stress and inflammasome activation can be a potential therapeutic approach in neurodegenerative diseases.

## 5. Role of Oxidative Stress in Pathological Protein Aggregates in Alzheimer’s and Parkinson’s Diseases

Regardless of the initial events that trigger AD and PD, both diseases show accumulation of aberrant protein aggregates. In AD, the appearance of aggregates of Aβ and tau is a requirement for the classification of the disease according to the 2018 NIA-AA research framework [149]. Similarly, the intraneuronal inclusions called Lewy bodies and Lewy neurites, immunopositive for α-syn, constitute one of the hallmarks in PD [150].

There is still controversy around whether protein aggregates may cause the disease or whether they are protective. In a recent work, Espay et al. [151] concluded that protein accumulation (Aβ and tau as amyloid plaques and neurofibrillary tangles in AD, and α-syn as Lewy bodies in PD) could be secondary to the initiation of the pathogenesis. In addition, these protein aggregates appear widespread in other conditions, such as the lysosomal disorder Sanfilippo syndrome [152] and the iatrogenic Creutzfeldt–Jakob disease [153]. Protein aggregates appear in post-mortem brains of neurologically normal individuals, especially in individuals aged over 90, suggesting these aggregates could have acted as a protective agent rather than as a toxic one [151]. Regardless of whether they can play a harmful or protective role on neurons, their formation is still unknown. In this part of the review we will focus on how oxidative stress may affect pathological aggregation of Aβ and α-syn.

Many studies have related oxidative stress to protein aggregation of α-syn and Aβ [154]. The main mechanisms through which oxidative stress leads to protein aggregation are oxidation, peroxidation, and nitration of proteins. It is well known that ROS induce oxidation in several molecules, including proteins. In PC12 cells treated with MPP^+^ and stimulated with low-intensity ultrasound, a significant drop in the α-syn aggregation was observed by inhibition of casein kinase 2-dependent ROS production [155]. ROS can also react with polyunsaturated fatty acids and produce damage via lipid peroxidation (LPO). When LPO products (malondialdehyde and 4-hydroxynonenal) are produced nonenzymatically, they are associated with cellular damage due to oxidative stress. In vitro studies have shown that these products facilitate the assembly of toxic α-syn oligomers, which were also increased in acidic conditions [156,157].

Another mechanism indicating that oxidative stress may alter α-syn aggregation is the peroxynitrite-dependent nitrative modification [131,158]. A proposed model suggests that nitrating and oxidant species induce fibrillization. In this sense, it has been seen that modifications of α-syn by RNS inhibit fibril formation (stabilizing the formation of lower molecular weight oligomers) [159,160] and result in protease-resistant, urea- and SDS-insoluble α-syn aggregates, considered responsible for cellular toxicity [158]. Interestingly, Hodara et al. demonstrated that while nitration of α-syn inhibits fibril formation, a coincubation of nitrated α-syn and unmodified α-syn resulted in incorporation of nitrated α-syn into the fibrils of unmodified α-syn [161]. It is suggested that this nitration acts very similarly to the A30P mutation in the *SNCA* gene that causes early onset PD, leading to the question of whether α-syn nitration is an early toxic modification or a later product of PD pathogenesis that further potentiates the disease [162].

In the case of Aβ, it has been also seen that peroxidase reaction induced dimeric forms of Aβ42. The residue implicated in this interaction is also tyrosine, increasing again the propensity of Aβ to aggregate [163,164].

Besides mitochondrial dysfunction and microglia activation, there are others sources of ROS production leading to protein aggregation. One of them is iron [165,166]. Its homeostasis is fundamental for biological processes such as mitochondrial respiration, myelin synthesis, and oxygen transport. Thus, alteration of its homeostasis and accumulation are observed in several neurodegenerative diseases and it has been shown to facilitate protein aggregation in these disorders [167]. This seems to be related to its protein–binding properties and the Fenton and Haber–Weiss reaction (Figure 2). The latter leads to protein aggregation by production of ROS (for review see [167]). Besides, α-syn and Aβ were found to affect iron metabolism and accumulation, suggesting a synergistic interplay to be implied in the detrimental states during the disease. Another metal to which α-syn is known to bind is copper, which accelerates its aggregation in vitro [168]. Like iron, it is also related to oxidative stress in neurodegenerative diseases when it binds to amyloidogenic proteins or peptides. The α-syn-Cu^2+^ complex can react with cellular species (e.g., dopamine, O_2_), increasing the oxidative environment, α-syn aggregation, and cellular damage [168]. With regard to Aβ42, copper also induces its aggregation and Aβ42-Cu^2+^ complexes lead to cell death in PC12 cells [169].

All these observations highlight oxidative stress as responsible for protein aggregation and consequent neurodegeneration in disorders such as AD and PD. Targeting this process might be used in future therapeutics strategies.

## 6. Epigenetic Regulation of Microglia Under Oxidative Stress Conditions in Neurodegeneration

Modulation of epigenetic mechanisms allows the alteration of cellular phenotypes without altering the genotype. The relationship established between redox-mediated mechanisms (via ROS or RNS) and modulation of epigenetic mechanisms is of a bidirectional sort, whilst depending on the environment, one may affect the other or vice versa.

There is growing interest in the ROS-mediated epigenetic regulation of the inflammatory response driven by different immune cells under pathological conditions. For instance, Kikuchi and colleagues established a relation between immune response and epigenetic regulation in the expression of one of the members of the NOX family [170]. The authors show that upon an inflammatory stimulus such as IFN-γ, monocytes promote the synthesis and recruitment of the histone acetyl transferase GCN5 into the gp91phox gene promoter, increasing the acetylation levels of H2BK16 and H3K9 surrounding such promoter. Also, the authors observed that the absence of GCN5 greatly decreased the expression of gp91phox and ROS production [170].

In another study, the authors showed that the origin of ROS elicited in tissues/cells following ischemia-reperfusion (I/R) can be linked to different sources, including both nonenzymatic (hemoglobin and myoglobin) and enzymatic sources. The enzyme systems most commonly used to explain the accelerated ROS production upon ischemia are xanthine oxidase, NOX, the mitochondrial electron transport chain, and uncoupled NOS [171].

In macrophages, the expression levels of megakaryocytic leukemia 1 (MKL1) are increased after cardiac-reperfusion injury [172]. The role of MKL1 in this type of injury is to recruit the histone acetyltransferase MOF and activate NOX transcription. Using MG149, a MOF inhibitor, it decreased the expression levels of *Nox1* and *Nox4* and attenuated cardiac ischemia-reperfusion injury in mice [172]. In microglia cells, the use of sodium butyrate as a histone deacetylate inhibitor modulates the levels of histone 3-lysine 9-acetylation (H3K9ac) that are surrounding different promoters of genes related to the inflammatory response. The use of sodium butyrate mitigates microglia-mediated neurotoxicity upon middle cerebral artery occlusion, decreasing the expression of *Tnfα* and *Nos2*, and upregulating the expression of the anti-inflammatory mediator *Il10* [173]. 

Another study showed that let-7c-5p directly targets the 3’-untranslated region of the caspase-3 mRNA, decreasing caspase-3 expression levels. This caspase-3 reduction regulates microglia activation [174,175], as it has been previously stated [176] (for review of the nonapoptotic role for caspase-3 and others in microglia see the review [7]). MicroRNA let-7c-5p is not the only microRNA that has been shown to modulate microglial activation under traumatic conditions. For instance, miR-203 was described as a negative regulator in ischemia, inducing microglia activation by targeting myeloid differentiation primary response 88 (MyD88) [177] upon ischemia. Also, miR-124 regulates microglial phagocytic activity in rodent models of spinal cord contusion injury [178]. Interestingly, lack of NOX2 decreased the expression of miR-155, a critical regulator of the inflammatory signaling [179,180], in a process mediated by IL-10.

In PD or AD there is a slow, long-term chronic inflammatory component, which starts even decades before the disease has been diagnosed. Aging is a predominant risk factor for neurodegenerative diseases where there is accumulation of DNA oxidative damage in mitochondria of microglia, which increase intracellular ROS production [181]. The effect of aging over the basal microglial activation shows, in general, an increase of basal levels of inflammatory cytokines [182]. This low but sustained production may have a profound impact on the brain aging process. One of the consequences of aging is the effect over the levels of Sirtuin-1 (SIRT1) in all tissues [183], a trend further highlighted in AD patients compared to control cases [184,185]. A recent report showed that an aging-dependent decrease in microglial SIRT1 has a causative role in tau-mediated memory deficits via IL-1β upregulation. This increase of IL-1β would be mediated through hypomethylation of specific CpG sites on IL-1β proximal promoter in human Tau^P301S^ (PS19) mice [186]. In a different study, the authors observed changes in the expression of the microglial histone H3K27me3 demethylase jumonji domain containing 3 (Jmjd3) with aging [187]. The authors showed how decreased levels of Jmjd3 lead microglia polarization into a supportive phenotype and an exacerbating microglia proinflammatory phenotype, inducing more neurodegeneration of the dopaminergic system in their MPTP mouse model.

## 7. Clinical Studies Using Anti-Inflammatory/Antioxidative Compounds

Taking into account the key role that inflammation and oxidative stress play in the development of neurodegenerative diseases, a great deal of interest has been devoted to developing possible neuroprotective agents for these diseases for the last 20 years. Some candidates have shown neuroprotective effects both in vivo and in vitro in models of PD, AD, HD, MS, or ALS. Therefore, a vast amount of clinical trials have been developed in an effort to find new treatments aimed to protect neurons and, thus, delay the progression of the disease (Table 1).

Currently, there are clinical trials using several natural antioxidants, such as resveratrol, vitamin D and E, polyphenols, lipoic acid, or Q10 coenzyme, among others. For instance, dietary regimes with high polyunsaturated fatty acid intake seem to reduce the frequency of relapses over two years in MS patients, although this intervention has no major effect on the main clinical outcome of the disease [212].

Another interesting compound is creatine. It is known that creatine improves oxidative stress, glutamatergic excitotoxicity, and apoptosis in both in vitro and in vivo models [217]. Since these mechanisms are involved in several neurodegenerative diseases, creatine supplementation has received a lot of attention as a neuroprotective strategy, with several promising studies in animal models of neurodegenerative diseases. This has led to a number of randomized clinical trials with oral creatine supplementation in patients with PD, HD, MS, and ALS. 

Laquinimod is an orally active immunomodulator that downregulates proinflammatory cytokine production in peripheral blood mononuclear cells. In the brain, laquinimod supresses astrocytic and microglial activation by modulating NFκB signaling [218]. It has been demonstrated that this compound has dual properties of immunomodulation and neuroprotection and is a potentially promising new oral disease-modifying therapy in clinical trials with MS and HD patients.

Moreover, epidemiological studies have consistently demonstrated that incidence of idiopathic AD and PD is about 50% lower in chronic users of nonsteroidal anti-inflammatory drugs (NSAIDs) or COX inhibitors than in age-matched nonusers [219,220].

Unfortunately, most of the clinical trials are not showing encouraging results, either because of the limitations of the studies or because of the poor efficacy of the proposed treatments. This low effectiveness could also be explained because they are being used at late stages of the neurodegenerative diseases.

Another compound with anti-inflammatory and antioxidant properties that deserves special attention is minocycline (7-dimethylamino-6-dimethyl-6-deoxytetracycline). This is a second generation, semi-synthetic tetracycline analogue active against a wide range of aerobic and anaerobic Gram-positive and Gram-negative bacteria [221]. This antibiotic has been mainly used for over 30 years in the treatment of acne vulgaris and some sexually transmitted diseases. Moreover, minocycline shows a good pharmacokinetic profile, a good safety record, and can easily pass through the blood–brain barrier due to its high lipophilic properties.

Most interestingly, besides its antibiotic effect, minocycline has been shown to have other properties including anti-inflammatory and antiapoptotic activities and inhibitory effects on proteolysis, tumor metastasis, and angiogenesis [222]. All these reasons have made minocycline a good candidate to treat diseases with an inflammatory basis, including rosacea, bullous dermatoses, neurophilic diseases, and sarcoidosis, among others [223,224,225].

Several proposed mechanisms of action of minocycline may be involved in its anti-inflammatory and antioxidant effects. These include: (i) inhibitory effects on the activities of key enzymes like iNOS and PLA2; (ii) reduction of protein tyrosine nitration because of its peroxynitrite-scavenging properties; (iii) inhibition of caspase-1 and caspase-3 activation; (iv) enhancement of BCL-2-derived effects, thus protecting the cells against apoptosis; (v) reduction of p38 MAPK phosphorylation; and (vi) inhibition of PARP-1 activity. The well-known ability of tetracycline to bind Ca^2+^ and Mg^2+^ may account for some of these biological activities, via the chelation of these cations and their transport into intracellular compartments (reviewed in [222]).

Taking into account the above mentioned anti-inflammatory/antioxidant properties of minocycline and its capacity to cross the blood–brain barrier, it could be useful in the treatment of neurodegenerative diseases in which inflammation and oxidative stress play a central role, such as PD, AD, MS, HD, or ALS. Therefore, minocycline has been used in several in vivo and in vitro models of these diseases with promising results [226,227,228], which has made this drug emerge as an attractive strategy to treat these neurological disorders.

Many clinical trials have been then performed in an effort to prove the efficacy of this treatment in the clinic (see Table 1). Hence, the potential neuroprotective activity of minocycline is currently undergoing phase II or III trial testing in AD, HD, PD, MS, and ALS patients [229]. Patients suffering these neurological disorders receive doses ranging from 200 to 400 mg/day; effectiveness, tolerability, and safety of these treatments are being studied for 6 to 24 months. These clinical trials are showing some promising results. Hence, the risk of conversion from a clinically isolated syndrome to MS was significantly lower with minocycline than with placebo, suggesting that minocycline will be considered for definitive phase III trials to determine if it alters the long-term progression of neurodegenerative diseases. For all these reasons, minocycline seems to be one of the most promising molecules for the treatment of neurodegenerative diseases, expected to give good results in the near future. However, factors such as safety, tolerability, activity, cost, and availability of this drug must be considered when comparing minocycline with other agents currently used in neurodegenerative diseases.

## 8. Conclusions

The identification of multiple microglia subtypes has certainly changed our view about their protective/deleterious roles in neurodegenerative diseases. The recent identification of DAM microglia should open new research strategies aimed at identifying different (even contradictory) roles of microglia under disease conditions. Two microglia receptors, TLRs and TREM2, drive main polarization states, proinflammatory and DAM. Ligands of these receptors fit well with the classical DAMPs, supported by the recent discovery of *Lgals3* (GAL3), a DAM gene highly upregulated in microglia clustering Aβ plaques. Other examples of multivalent ligands are APOE and Aβ itself through its different conformational states. Consequently, a cross-talk between these two main microglia polarization states is expected to increase heterogeneity. Different DAM subtypes are actually supported by WGCNA, including the existence of pro-oxidant DAM.

In summary, the unique plasticity of microglia upon different kinds of stimuli has become a great challenge in the field. To uncover the broad panel of polarization states of microglia that may exist and/or coexist during the onset and progression of different neurodegenerative disorders is an unmet clinical need that may help to develop novel immunotherapeutic strategies. 

## Figures and Tables

**Table 1:** Clinical trials using anti-inflammatory/antioxidant drugs for the prevention or improvement of neurodegenerative diseases.

Disease	Compound	Effects	Reference
**Huntington´s disease**	Minocycline	Trial in progress	CN-01508775
	Laquinimod	Trial in progress	CN-01364776
	AZD3241	Trial in progress	[188]
	Riluzole	Trial in progress	CN-01513126
	Antioxidants	Trials in progress	CN-00120692;CN-00150820
	Fenifibrate	Trial in progress	CN-01574117
	Memantine	Trial in progress	CN-01533522
	Creatin	Trial in progress	NCT00592995
**Parkinson’s disease**	Green tea	Trial in progress	NCT00461942
	Polyphenols	Trial in progress	NCT00461942
	NSAIDs	May reduce the risk of developing PD	[189]
	Q10 coenzyme	Trial in progress	NCT00076492
	Pramipexole	Improved motor impairments and disability	[190]
	Pergolide	Improved motor impairments and disability	[191]
	Bromocriptine	No prevention in the onset of motor complications	[192]
	Cabergoline	Improved motor impairments and disability	[193]
	Ropinirole	Improved motor impairments and disability	[194]
	MAO B inhibitors	Reduced rate of motor fluctuations	[195]
	Minocycline	Phase II trial	[196]
	Polyphenols	Trials in progress	NCT01001637; NCT00743743; NCT00164749
	Vitamin E	No evidence	[197]
	Nicotine	No results available	[198]
	Piracetam	No benefit was shown	[199]
	Vitamins and minerals	Evidence very limited	[200]
	Masitinib	Trial in progress	CN-01867921
	NSAIDs	Sparing effect ranging between 36% and 80%	[201,202,203]
	Minocycline	Trial in progress	CN-01797324; CN-01847636
**Multiple sclerosis**	Minocycline	Decreased risk of conversion from a clinically isolate syndrome to multiple sclerosis	[204]
	Statins	No convincing evidence	[205]
	Dimethyl Fumarate	Moderate-quality evidence of decreased relapses frequency	[206]
	Laquinimod	Low-level evidence as a disease-modifying therapy	[207]
	Amantadine	Poorly documented	[208]
	Fingolimod	Reduced inflammatory disease activity	[209]
	Vitamin D	No benefit	[210]
	Carnitine	Insufficient evidence	[211]
	PUFAs	Reduced relapses frequency	[212]; CN-01579873
	Lipoic acid	Trials in progress	CN-01518782; CN-01878397; CN-01266929
	Epigallocatechin gallate	Trial in progress	CN-01794896
	Q10 coenzyme	Trials in progress	CN-00866966; CN-01136521
	Melatonin	Trial in progress	CN-01114089
	Crocin	Trial in progress	CN-01836077
	Curcumin	Trial in progress	N-01896018
	Alpha-tocopherol	Trial in progress	CN-00912436
	ω3 fatty acids	Trial in progress	CN-01650553
	Fish oil	Trial in progress	CN-00982838
	Creatin	Trial in progress	CN-01502285
	NSAIDs	Trial in progress	CN-01010452
**Amyotrophic lateral sclerosis**	Vitamin E	Trial in progress	CN-00442211
	Creatin	No effect on survival	[213]
	Minocyclin	Trials in progress	CN-01616430; CN-01259752; CN-01381473
			CN-00730827
	Riluzole	Probably prolongs survival	[214]
	Celecoxib	Trial in progress	CN-00566124
	Curcumin	Might improve survival probability	[215]
	Antioxidants	Insufficient evidence of efficacy	[216]

Some of these compounds are specifically included in clinical trials due to their anti-inflammatory or antioxidative properties. Abbreviations: MAO B, monoamine oxidase B; NSAIDs, nonsteroidal anti-inflammatory drugs; PUFAs, polyunsaturated fatty acids.

## Appendix A

Box A1 Tools for studying oxidative stress in vivo/in vitro. The critical relevance of oxidative stress in neurodegenerative diseases exposes the need for reliable biomarkers of redox status to monitor disease stages and progression [230]. Biomarkers and methods for redox status monitorization can be divided in two groups:
*(a) DIRECT METHODS*
The direct quantification of ROS/RNS may reflect the pathological process in some diseases, but the natural high reactivity and short lifespan of the species make its direct measurement extremely difficult in the pathogenic focus [231] and is currently limited to in vitro applications. Direct methods include electron spin resonance, fluorescence magnetic resonance, flow cytometry, mass spectrometry techniques, or chemiluminiscent probes [232,233,234].
*(b) INDIRECT METHODS*
Indirect methods are based on the different products of the reactions of ROS/RNS with the main biomolecules: proteins, lipids, and nucleic acids.
**(i) Protein oxidation**
3. Nitrotyrosine 3-nitrotyrosine (3-NT) can be obtained mainly from tyrosine residues in proteins through the chemical conversion by tetranitromethane and peroxynitrite [235,236]. The methods used for detection include immunochemical and chromatographic procedures, including liquid chromatography using ultraviolet, fluorescence, and electrochemical detection and variants like liquid chromatography–mass spectrometry (LC–MS) or gas chromatography–mass spectrometry (GC–MS) [236]. Currently, the most used methods for measuring 3-NT are immunochemical methods including ELISA because it is a cheap, simple, and suitable assay [237]. Glutathione The tripeptide GSH is synthesized in two steps from the amino acids glutamate, cysteine, and glycine, catalyzed by the enzyme glutamate cysteine ligase. GSH is used as a biomarker because it is the most abundant endogenous intracellular antioxidant, so any condition associated with excessive ROS will decrease GSH levels or the GSH/GSSG ratio [238]. In addition, GSH is able to react with a reactive cysteine residue and form a disulphide bridge, giving rise to GSSG, which is also used as a biomarker [239]. Although there are several widely used methods to measure GSH and GSSG levels, like Western blotting, MS techniques, ELISA, or enzyme assays, the most sensitive method is high performance liquid chromatography (HPLC) [240,241]. Protein carbonyls This biomarker can be detected after its derivatization, resulting in a stable product (DNP) [242]. To measure this biomarker, HPLC, proteomic techniques [243], and, for more specificity, techniques based on the use of anti-DNP antibodies, like ELISA or Western blot [242] could be used. Advanced glycation end products (AGEs): The AGEs are proteins or lipids that become glycated as a result of exposure to sugars [244]. The most used methods to identify these adducts are mass spectrometry-based techniques [245], immune-based methods, or HPLC [246]. Advanced oxidation protein products (AOPP) AOPPs are created during oxidative stress through the reaction of plasma proteins with chlorinated oxidants. 3-chloro-tyrosine is one of the main products of the reaction [231] and could be detected by mass spectrometry [247,248].
**(ii) Lipid oxidation**
Malondialdehyde and 4-hydroxy-2-nonenal Malondialdehyde (MDA) and 4-hydroxy-2-nonenal (HNE) are the best-known end products of lipid oxidation [249]. To detect HNE directly, HPLC, GC-MS, or immunological techniques using specific anti-HNE antibodies [249] could be used. On the other hand, MDA could react with thiobarbituric acid to give an adduct, which can be measured by colorimetric or fluorimetric assays. However, HPLC combined with UV or fluorescence is normally selected for its measure [250]. F2 isoprostanes and isolevuglandins Isoprostanes (IsoPs) are a family of stable prostaglandin-like compounds generated by the free radical-induced peroxidation of arachidonic acid [251]. The most useful as biomarkers are F2-IsoPs. HPLC/GC-MS is the most reliable method for its detection [252]. Isolevuglandins (IsoLGs) are products from the oxidation of arachidonic acid. They can be measured owing to structural modifications through liquid chromatography/tandem mass spectrometry methods (LC/MS/MS) [253].
**(iii) Nucleic Acids oxidation**
Nucleic acids are highly susceptible to oxidation and by this process oxidizing agents generate many modifications in DNA and RNA. If these modifications are not repaired, they could potentially lead to mutagenesis [254]. The main mutagenic lesions of nucleic acids caused by free radical are the 8-oxo-7, 8-dihydroguanine (8-oxoGua), and 8-oxo-7, 8-dihydroguanosine (8-oxodG) [231]. The levels of 8-oxoGua and 8-oxodG can be measured by HPLC-MS/MS, but limitations of this technique make the evaluation of low damage levels not reliable [254,255]. In contrast, ELISA assays are useful for measuring low levels of damage, but these methods are less specific compared to HPLC/MS-MS [256,257]. Overall, a more reliable method to measure low levels of damage relies on enzymatic methodology, which uses excision DNA repair enzymes formamidopyrimidine DNA glycosylase (Fpg), endonuclease III (EndoIII), or 8-hydroxyguanine glycosylase 1 (hOGG1) [230,255].
